# Cysteamine Eye Drops in Hyaluronic Acid Packaged in Innovative Single-Dose Systems, Part II: Long-Term Stability and Clinical Ocular Biopermanence

**DOI:** 10.3390/pharmaceutics15112589

**Published:** 2023-11-05

**Authors:** Ana Castro-Balado, Andrea Cuartero-Martínez, Hugo Pena-Verdeal, Gonzalo Hermelo-Vidal, Anja Schmidt, Belén Montero, Manuela Hernández-Blanco, Irene Zarra-Ferro, Miguel González-Barcia, Cristina Mondelo-García, María Jesús Giráldez, Eva Yebra-Pimentel, Francisco J. Otero-Espinar, Anxo Fernández-Ferreiro

**Affiliations:** 1 Pharmacy Department, University Clinical Hospital of Santiago de Compostela (SERGAS), 15706 Santiago de Compostela, Spain; ana.castro.balado@gmail.com (A.C.-B.); irene.zarra.ferro@sergas.es (I.Z.-F.); miguel.gonzalez.barcia@sergas.es (M.G.-B.); crismondelo1@gmail.com (C.M.-G.); 2 Clinical Pharmacology Group, Health Research Institute of Santiago de Compostela (IDIS), 15706 Santiago de Compostela, Spain; andrea.cuartero@rai.usc.es (A.C.-M.); zalohermelo@gmail.com (G.H.-V.); 3 Pharmacology, Pharmacy and Pharmaceutical Technology Department, Faculty of Pharmacy, University of Santiago de Compostela (USC), 15782 Santiago de Compostela, Spain; 4 Department of Applied Physics (Optometry), Faculty of Optics and Optometry, University of Santiago de Compostela (USC), 15782 Santiago de Compostela, Spain; hugo.pena.verdeal@usc.es (H.P.-V.); mjesus.giraldez@usc.es (M.J.G.); eva.yebra-pimentel@usc.es (E.Y.-P.); 5 Optometry Group, Health Research Institute of Santiago de Compostela (IDIS), 15706 Santiago de Compostela, Spain; 6 Group of Polymers, Physics and Earth Sciences Department, Campus Industrial de Ferrol (CIF), CITENI, Escuela Politécnica de Ingeniería (EPEF), Universidade da Coruña, C/Mendizabal s/n, 15403 Ferrol, Spain; a.schmidt@udc.es (A.S.); belen.montero@udc.es (B.M.); 7 Microbiology Department, University Clinical Hospital of Santiago de Compostela (SERGAS), 15706 Santiago de Compostela, Spain; manuela.hernandez.blanco@sergas.es

**Keywords:** cysteamine, cystinosis, eye drops, ophthalmic administration

## Abstract

Background: Cystinosis is a rare genetic disorder characterized by the accumulation of cystine crystals in several tissues and organs causing, among others, severe eye symptoms. The high instability of cysteamine eye drops makes it difficult to develop formulations with an acceptable shelf life to be prepared in hospital pharmacy departments. Previously, a new compounded formulation of cysteamine eye drops in hyaluronic acid (HA) packaged in innovative single-dose systems was developed. Methods: Long-term stability at −20 °C of this formulation was studied considering the content of cysteamine, pH, osmolality, viscosity, and microbiological analysis. The oxygen permeability of single-dose containers was also studied and an ocular biopermanence study was conducted in healthy volunteers measuring lacrimal stability and volume parameters. Results: Data confirm that cysteamine concentration remained above 90% for 120 days, all parameters remaining within the accepted range for ophthalmic formulations. The permeability of the containers was reduced over time, while ocular biopermanence was maintained despite the freezing process and storage time. Conclusions: 0.55% cysteamine hydrochloride formulation in HA and packaged in single-dose containers preserved at −20 °C is stable for 120 days protected from light, presenting high potential for its translation into clinical practice when commercial presentations are not available.

## 1. Introduction

Cystinosis is a rare lysosomal disease characterized by intralysosomal accumulation of cystine crystals, causing damage to various organs and tissues, particularly kidneys and eyes [1,2]. This accumulation process is caused by mutations in the CTNS (17p13) gene, which encodes cystinosin, the transporter that carries cystine out of lysosomes [3,4]. Cysteamine lowers intracellular levels of cystine by forming a cysteamine–cysteine mixed disulfide, which resembles lysine and leaves the lysosome using alternative cationic transporters [5,6]. Cysteamine reacts easily with oxygen to form a disulfide called cystamine, ineffective in the treatment of cystinosis [7,8,9]. The fact that cystinosis is a rare disease arouses limited interest in the pharmaceutical industry, so developments in recent years have been scarce. To date, oral formulations have been marketed, such as Cystagon^®^ and Procysbi^®^ [10,11,12], with low ophthalmic cysteamine bioavailability, leading to the development of ophthalmic formulations. Cystaran^®^ 0.44% eye drops, received the U.S. Food and Drug Administration (FDA) approval in 2012 as an orphan drug [13] and, later, Cystadrops^®^ 0.55% was approved by the European Medicines Agency (EMA) and the FDA in 2017 and 2021, respectively.

Commercial cysteamine eye drops are not always available in all countries as there are sometimes delays to required procedures and licensing. In this case, Hospital Pharmacy Departments (HPD) are responsible for the elaboration of ophthalmic cysteamine formulations as an alternative therapeutic option [14]. Prior to translation to clinical practice, characterization and stability studies are required to guarantee adequate galenic properties, being its extreme instability the main problem for their development [14,15]. Oxygen removal, adding antioxidants, reducing the pH, and lowering the temperature in storage, have been shown to effectively increase cysteamine stability [16,17,18,19]. Choosing a suitable vehicle is also important, as higher viscosity enhances precorneal residence time and reduces nasolacrimal drainage, increasing the ocular bioavailability of the active ingredients and reducing the frequency of administration [20]. In this regard, sodium carboxymethylcellulose was the main advantage provided by Cystadrops^®^ over Cystaran^®^, offering greater ocular surface permanence and more convenient dosage. This excipient provides high viscosity, improving residence time on the ocular surface and allowing its administration four times a day instead of once every hour as with Cystaran^®^ [21,22,23]. Hyaluronic acid (HA) as a vehicle has great advantages due to its viscosity, and mucoadhesive and biocompatible properties [24,25]. Bearing this in mind, a new compounded formulation of 0.55% cysteamine hydrochloride eye drops in HA packaged in innovative single-dose systems has been previously developed by our group [26]. Results showed that this formulation stored at 2–8 °C was stable for less than a week, while when stored at −20 °C, a stability of at least 30 days was reached. Saturation of the solution with nitrogen showed no additional stability. In addition, ocular biopermanence preclinical studies were conducted using positron emission tomography (PET), concluding that the ocular surface permanence offered by the HA is not affected by the reduction in viscosity derived from freezing process, where a half-life of 31.11 min for a HA formulation stored for 30 days at −20 °C was obtained, compared with 14.63 min for 0.9% sodium chloride eye drops [26].

The development of this formulation was a breakthrough for patients and HPD. Knowing its maximum period of stability would allow its translation into clinical practice, allowing the treatment to be dispensed for longer periods and reducing the number of visits to the hospital and the workload of HPD. The present study aimed to determine the previously developed cysteamine compounded formulation [26]: (1) the maximum stability period in terms of chemical, physical, and microbiological stability, as well as to evaluate packaging oxygen permeability and how it is affected by the freeze-thaw process; and, (2) the clinical ocular surface permanence in terms of tear stability and volume in healthy volunteers to confirm the previous preclinical results.

## 2. Materials and Methods

### 2.1. Materials

Cysteamine hydrochloride (Cysteamine purity 97%) was obtained from Apollo Scientific^®^ (Stockport, UK). Balanced salt solution (BSS^®^) was purchased from Alcon^®^ (Geneva, Switzerland) and Aquoral^®^ (AQ) eye drops (0.4% *w*/*v* % HA) were purchased from Esteve (Barcelona, Spain). For the mobile phase, 1-heptanesulphone acid sodium salt was obtained from Sigma Aldrich^®^ (St. Louis, MO, USA), acetonitrile UHPLC-MS grade and acetic acid were purchased from VWR Chemicals^®^ (Radnor, PA, USA), and ultrapure water was obtained from MilliQ, Merck Millipore^®^ (Madrid, Spain). As the packaging material, COL Eye Drops System was obtained from Biomed Device^®^ (Modena, Italy), being used as single-dose containers.

### 2.2. Methods

#### 2.2.1. Elaboration and Packaging of Cysteamine Sterile Solutions

As described in our previous work [26], cysteamine was dissolved in 10% *v*/*v* BSS^®^ with magnetic stirring, and then sufficient AQ was added diluent to achieve a cysteamine hydrochloride concentration of 0.55%. Sterilizing filtration was performed with a 0.22 µm membrane filter (vacuum-driven bottles FPR204250 PES 0.22 µm; Biofil, Alicante, Spain) under vacuum [26]. Cysteamine solutions were elaborated in triplicate. Under sterile conditions in a horizontal laminar flow cabinet, single-dose systems were filled with 30 mL of each formulation by first evacuating the air contained inside using the coupled syringe, connections, and filters according to the manufacturer’s instructions [27,28]. Finally, each single-dose container was sealed with a Qseal^®^-opti heat-sealing equipment (Conroy Medical^®^, Upplands Väsby, Sweden) and individually checked.

#### 2.2.2. Long-Term Stability Study

Once the formulations were prepared, single-dose vials were stored protected from light in a freezer (−20 °C) for 120 days. Every 30 days, vials were thawed, and physical and chemical properties were determined, stopping the study when the cysteamine concentration fell below 90% of the initial concentration. Studies were performed in triplicate. All samples were kept at room temperature for at least 30 min once thawed prior to analysis to avoid measurement errors due to temperature variations. Table 1 shows the experimental design of all studies performed for 120 days.

#### 2.2.3. Cysteamine Quantification

Cysteamine was quantified by ultra-high-performance liquid chromatography (UHPLC) as described in our previous work [26], with a UHPLC coupled to photodiode array detection (PDA), using an ACQUITY UPLC H-Class System (Waters^®^, Milford, MA, USA) with ACQUITY PDA detector (Waters^®^). An ACQUITY^®^ BEH C18 column (2.1 × 50 mm, 1.7 µm, Waters^®^) at a temperature of 45 °C was employed. The mobile phase was a mixture of aqueous 4 mM sodium 1-heptane sulfonate: acetonitrile, in gradient mode as previously described [26]. The wavelength used for the detection of cysteamine was 215 nm, respectively. Data were collected and processed with Empower 3 Software (2002–2019 Waters^®^) Application Manager.

All test samples were diluted 1:50 with 0.1% acetic acid and analyzed by UHPLC setting the injection volume to 5 µL.

#### 2.2.4. Determination of pH and Osmolality

To determine the pH of the formulation, a BasiC20^®^ pH meter (CRISON^®^, L’Hospitalet de Llobregat, Spain) was used and, to measure the osmolality, 150 µL aliquots of the formulation were introduced in a cryofreezing point osmometer (OsmoSpecial 1; Scharlab^®^, Sentmenat, Spain). Each assay was performed in triplicate on day 0 (unfrozen formulation) and at days 30, 60, 90 and 120 post-freezing (Table 1).

#### 2.2.5. Viscosity Tests

The viscosity of cysteamine in HA eye drops was determined in triplicate on days 0 (unfrozen formulation) and at days 30, and 120 post-freezing with a rotational viscometer (ViscoQC 300 with PTD 80 Peltier Temperature; Anton Paar^®^, Madrid, Spain) (Table 1). For this, 2 mL of each formulation was introduced into the equipment, and the measurement was carried out at 25 °C and 100 revolutions per minute (rpm).

#### 2.2.6. Microbiological Stability

Each replicate was analyzed on day 0 (unfrozen formulation) and at days 60 and 120 post-freezing to determine microbiological stability (Table 1). Aliquots of 1.5 mL were added to plates containing blood agar, Sabouraud agar, and fluid thioglycolate medium. Afterward, media were incubated aerobically at 37 °C; thioglycollate for 10 days, and blood and Sabouraud agar plates for 48 h. Sabouraud agar plates were subsequently incubated for 13 days in aerobiosis at room temperature.

#### 2.2.7. Single-Dose Oxygen Transmission Rate

To determine whether oxygen transmission through the single doses is affected after the freeze-thaw process, measurements were made of the oxygen transmission rate (OTR) at day 0 (unfrozen formulation) and at days 30 and 120 post-freezing (Table 1). OTR of the walls of the single-dose vials was measured using an OX-TRAN, 1/50 G, Mocon system (Mocon Inc., Minneapolis, MN, USA) according to the specifications described in ASTM D-3985 [29]. A single sample was used for analysis on each of the test days. Single-dose containers were previously thawed and emptied and subsequently cut to obtain a film with a thickness of 1.00 ± 0.01 mm, measured with a digital precision gauge model 3050 from Baxlo (Barcelona, Spain). Samples were introduced into the equipment cell using a mask with a test area of 5 cm^2^. The temperature of the cell was 23 °C. One side of the film was exposed to a stream of dry oxygen at a relative humidity of 1% and a pressure of 32 psi, initiating the diffusion of oxygen molecules into the film. OTR was calculated by counting the molecules passing through the membrane every 20 min. The permeated molecules were delivered to the counting section of the device, equipped with a coulometric sensor, by dry nitrogen (carrier gas), which was continuously purged at 32 psi throughout the test. The tests continued and a steady line of transmission rate in continuous mode was obtained (OTR in cm^3^ m^−2^ day^−1^).

#### 2.2.8. Ocular Surface Permanence Study in Healthy Volunteers

In order to demonstrate the behavior of the formulation on the ocular surface, a study of tear meniscus height (TMH) and non-invasive tear keratograph breakup time (NIKBUT) was performed with OCULUS Keratograph 5M^®^ (Oculus, Wetzlar, Germany) and Oculus TF-Scan module (Oculus, Wetzlar, Germany). TMH is the distance between the uppermost edge of the lower eyelid and the upper limit of the lacrimal meniscus, while NIKBUT reflects the time elapsed from the last complete blink to the first tear film breakup [30,31]. An ocular surface permanence clinical study was conducted in 10 healthy volunteers (20 eyes) at the Optometry Clinic of the Optometry Faculty of the Universidade de Santiago de Compostela. The inclusion and exclusion criteria are listed in Table S1 of the supplementary material. To mitigate the influence of diurnal variations in tear parameters or interexaminer influence, all measurements were conducted within the same time window (09:00–12:00 a.m.) by the same investigator across all sessions and patients [32,33,34], and with environmental conditions controlled and maintained under similar conditions of temperature (20–23 °C) and humidity (50–60%). The study protocol adhered to the tenets of the Declaration of Helsinki and was approved by the Institutional Review Board/Ethics Committee of the Ethical Committee of Clinical Research of Galicia (2019/204).

Participants underwent three separate sessions at day 0 (unfrozen formulation) and at days 30 and 120 post-freezing in which a baseline measurement of the TMH and subsequently of the NIKBUT was performed before the instillation of the formulation. Subsequently, 15 µL of the freshly made formulation (on day 0) or thawed formulation (on days 30 and 120) were instilled with a micropipette. TMH and NIKBUT were examined at 2, 5, 10, 15, and 30-min post-administration using Keratograph 5M^®^. For TMH measurements, participants were asked to blink normally and then leave the eyes open to take the image of the inferior lacrimal meniscus. In the case of the NIKBUT analysis, participants were instructed to blink 2 times and try to keep their eyes open as long as possible. The light source chosen was visible and infrared for TMH and NIKBUT evaluation, respectively. TMH and NIKBUT measurements were carried out in triplicate. TMH was measured manually with a ruler integrated into the instrument software. Data were collected by a different investigator than the one who later analyzed them to avoid bias.

### 2.3. Statistical Analysis

To establish the expiry date of the compounded formulations the Pharmaceutical Codex was used, setting a reduction of ≥10% of cysteamine with respect to the initial concentration [35]. The degradation rate (K), shelf-life (t_90_) and determination coefficient (R^2^) were calculated. Changes in pH and osmolality were considered unacceptable if their values exceeded the acceptance criteria for ophthalmic applications [36,37]. Microbiological stability was considered acceptable when no microbial growth occurred in the cultured samples.

The results of the different assays were plotted using Graph Pad Prism^®^ v.9.0.1 software (GraphPad Software, San Diego, CA, USA). The significance value was set at an α ≤ 0.05 for all statistical tests. Before analysis, the normal distribution of the data was checked using the two-way ANOVA test and the differences between results on devices were assessed using the ANOVA for paired measurements [38]. Mean and standard deviation (SD) were used to describe the measurements in lacrimal parameters.

## 3. Results

### 3.1. Physicochemical Stability

**Figure 1:**
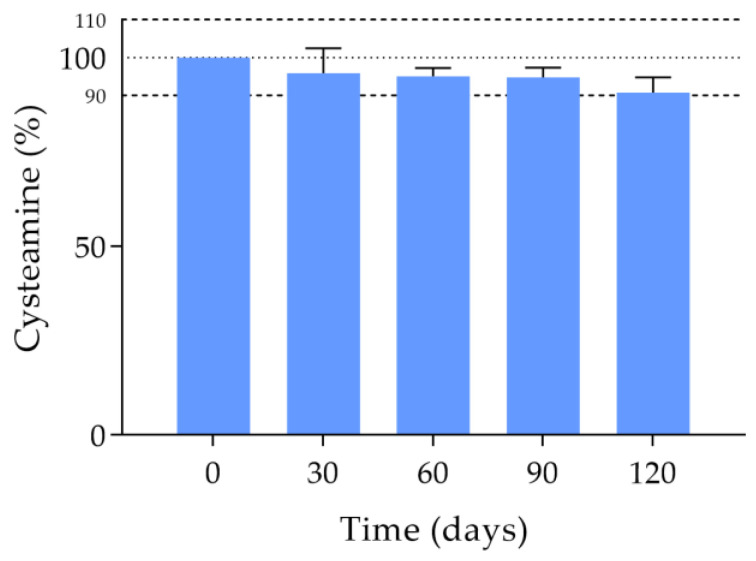
Cysteamine content (%) with respect to day 0 of the cysteamine compounded formulation stored at −20 °C for 120 days protected from light at day 0 (unfrozen formulation) and days 30, 60, 90 and 120 post-freezing.

The amount of cysteamine remaining in the single-dose containers throughout the study is shown in Figure 1. The concentration of cysteamine in the formulation remained above the stability limit (>90%) until day 120 after freezing. Since at this point the concentration was very close to 90%, it was decided to terminate the stability study, since at later times the formulation would no longer be stable. The degradation rate (K) and shelf-life (t_90_) were 0.07729%·day^−1^ and 137.14 days, respectively, with a determination coefficient (R^2^) of 0.9281.

**Figure 2:**
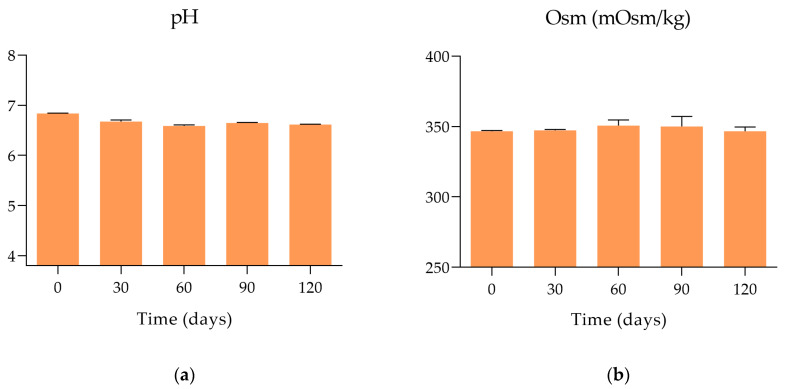
Variation in pH and osmolality (mOsm/kg) of the cysteamine formulation measured at day 0 (unfrozen formulation) and at days 30, 60, 90 and 120 post-freezing: (**a**) pH; (**b**) osmolality.

The variation of pH values throughout the study is depicted in Figure 2a. From these data it is possible to confirm that pH remains constant for 120 days after freezing, hovering around 6.6–6.8. During the study period, the osmolality of the formulations remained within ±5% of the initial value, approximately 350 mOsm/kg (Figure 2b). These results allow us to demonstrate that a longer period of storage does not affect these two physical parameters of the formulation.

**Figure 3:**
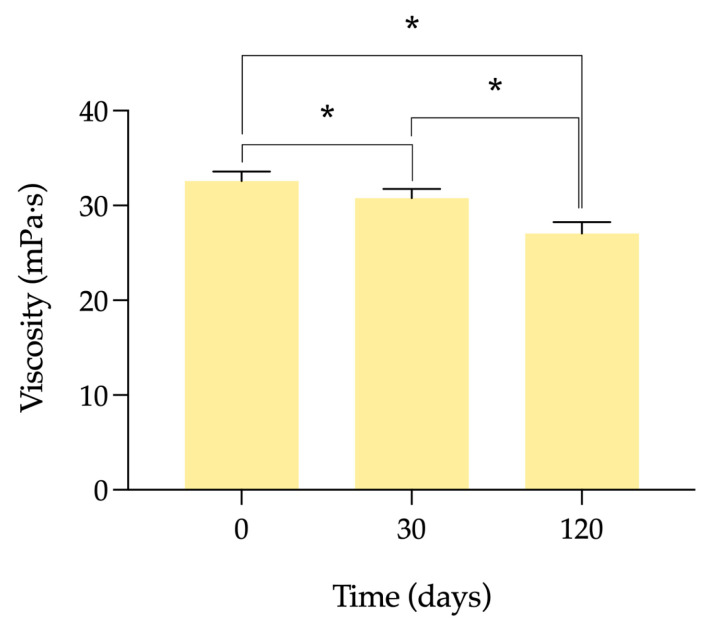
Viscosity of the cysteamine compounded formulation values measured at day 0 (unfrozen formulation) and at days 30 and 120 post-freezing. Measurements were made at 25 °C and 100 revolutions per minute (rpm). * statistical significance *p* < 0.05.

The viscosity results of the formulation at different times are shown in Figure 3. As was seen in the previous work, these values are around 30 mPa·s. As the time elapses during which the formulation is frozen, the viscosity decreases. This reduction is statistically significant when viscosity at day 0 and after 30 days frozen is compared (α = 0.0041), between day 0 and after 120 days after freezing (α < 0.0001), and between days 30 and 120 after freezing (α < 0.0001).

Furthermore, no macroscopic changes (color, turbidity, precipitation, etc.) were observed throughout the study period.

### 3.2. Microbiological Stability

Microbial growth control ensures quality control of a drug product, as it guarantees the sterility of the formulation and thus aseptic conditions during the manufacturing process. In this study, adequate elaboration and preservation of the eye drops during the study was observed as the presence of microorganisms was not detected in any of the samples examined.

### 3.3. Single-Dose Oxygen Transmission Rate

**Figure 4:**
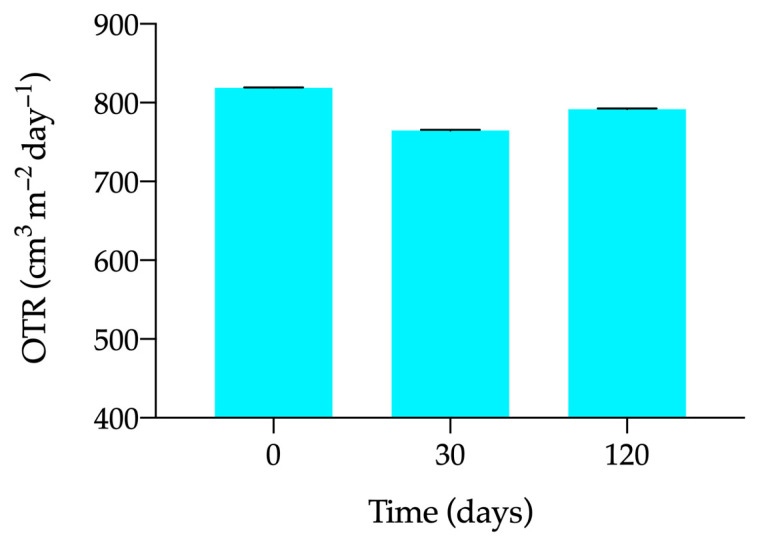
Oxygen transmission rate variation of the single-dose vials on days 0 (unfrozen formulation), 30 and 120 post-freezing.

Data show that the oxygen transmission rate decreases slightly over time under freezing conditions (Figure 4), so freezing seems to favor the structure of the material to remain rigid, preventing the gas from permeating through it without creating breaks or pores through which the O_2_ molecule can pass.

### 3.4. Ocular Surface Permanence Studies in Healthy Volunteers

A group of 10 healthy volunteers (20 eyes), three men and seven women, with an age of 30.5 ± 4.7 years (24–38) participated in the study, meeting all the inclusion criteria and none of the exclusion criteria.

**Figure 5:**
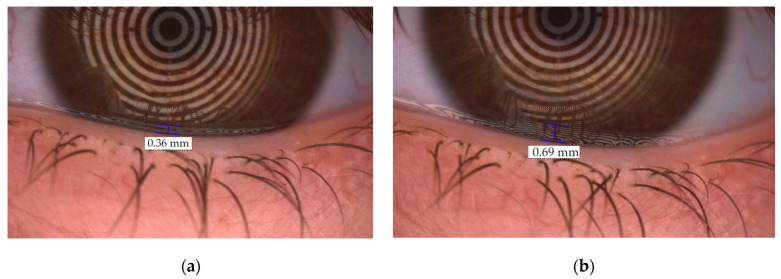
Vertical measurement of TMH in one of the healthy volunteers. (**a**) TMH before the instillation; (**b**) TMH 2 min after the instillation of the 30-day frozen formulation.

**Figure 6:**
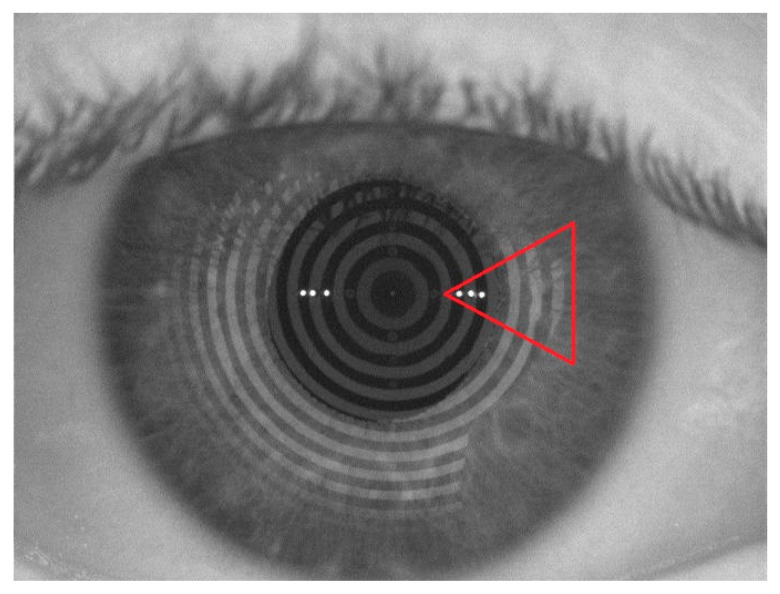
Representative image of NIKBUT. The red mark indicates the disruption on the tear film when breakup time was registered.

TMH and NIKBUT were measured in healthy volunteers on day 0 with the unfrozen formulation, and after freezing the formulation for 30 and 120 days. Measurements were performed at baseline without instillation and after instillation for 30 min. Figure 5 shows changes in TMH before (a) and after the instillation (b), and Figure 6 is a representative image of lacrimal rupture when breakup time is measured.

**Figure 7:**
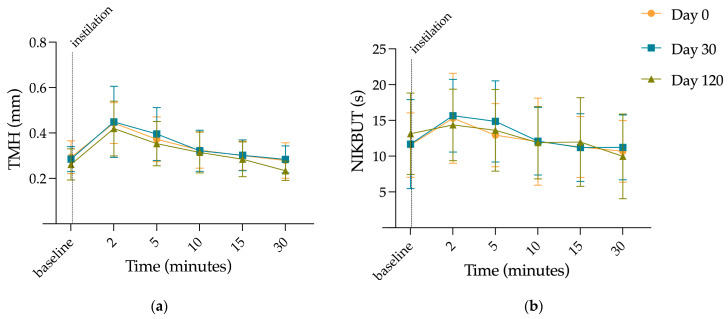
Mean values of the measurement time-points at different conditions. Day 0: unfrozen formulation; Day 30: 30-day frozen formulation; Day 120: 120-day frozen formulation. (**a**) Tear meniscus height (TMH); (**b**) Non-invasive keratograph tear breakup time (NIKBUT).

The mean TMH value for baseline measurements were 0.28 ± 0.066 mm, with a subsequent increase at 2 min after instillation with respect to basal to 0.474 ± 0.090, 0.449 ± 0.157, and 0.420 ± 0.121 for the unfrozen formulation, 30-day frozen formulation and 120-day frozen formulation, respectively. From that moment on, there was a decrease in the TMH, until reaching mean values similar to the baseline between minutes 10 and 15, relative to the elimination of the formulations due to physiological ocular eye wash (Figure 7a, and Table S2 of the supplementary material). This behavior was observed in a similar way in the three formulations. A comparison of these TMH values at different times (2, 5, 10, 15, and 30 min) was made between the unfrozen prepared formulation (day 0), and the 30-day and 120-day post-freezing formulation. Only differences between 30-day frozen eye drops and 120-day frozen eye drops at min 30 were found, with a mean TMH value of 0.2835 ± 0.059 mm for the formulation thawed at day 30, versus 0.2338 ± 0.043 mm for the formulation thawed at day 120 (Tukey, α = 0.0320).

Regarding the NIKBUT analysis, the administration of all formulations showed an increase at early test times compared to baseline values, relative to the residence of HA on the corneal surface after instillation, similar to what was previously observed for the TMH (10.89 ± 3.498 s at baseline versus 15.31 ± 6.292 s for unfrozen formulation; 11.69 ± 6.228 s at baseline versus 15.65 ± 5.083 s for 30-day frozen formulation; and 13.14 ± 5.690 s at baseline versus 14.36 ± 5.001 s for 120-day frozen formulation). This parameter was not altered in the comparison of formulation prior freezing and post-freezing formulations at all times (Tukey, all ≥ 0.9999, Figure 7b, Table S3 of the supplementary material). This was followed by a progressive decrease close to baseline levels related to the physiological processes of tear drainage and ocular eye wash.

## 4. Discussion

Cystinosis is a rare genetic disease in which cystine crystals accumulate in the lysosomes of certain tissues such as kidneys, pancreas, and eyes [39]. Although there are a couple of presentations marketed for the treatment of ocular cystinosis by the topical ophthalmic route (Cystagon^®^ and Cystadrops^®^), there are circumstances in which these treatments are not available. Therefore, HPDs continue to play a very important role in offering an alternative using pharmaceutical compounding [14]. To ensure adequate galenic characteristics and to assign a period of validity it is necessary to carry out exhaustive characterization studies prior to translation into clinical practice.

The limited stability of cysteamine in solution has led to the search for different approaches to prevent its oxidation. One of the proposals is to lower storage temperature conditions [8,19]. Permanent storage of cysteamine eye drops in the refrigerator is not feasible due to the frequency of instillation, which would require portability throughout the day by the patient under these conditions. Freezing is also not feasible when multidose containers are used since complete thawing of the entire solution would be necessary for each administration. To address these problems, we opted for packaging the developed cysteamine ophthalmic compounded formulation in single-dose containers [26]. In the present work, the chemical stability of cysteamine in these containers stored for 120 days at −20 °C was studied, from which it was found that the concentration with respect to day 0 remains above 90% during all the study time.

Numerous stability studies of cysteamine eye drops have been published, most of them carried out at room temperature and/or under refrigerated conditions [14,15,40,41]. Reda et al. studied the stability of a 0.44% aqueous solution of cysteamine hydrochloride for a year at room temperature and in a freezer [16]. This formulation contained only 61.6% cysteamine with respect to the initial when stored at room temperature for one week. In contrast, when storage was carried out under frozen conditions, cysteamine was maintained at 85.1% after 1 week, which would rule out its use (<90% of active ingredient) [35]. However, after 52 weeks in the freezer, the concentration did not fall sharply, remaining at 85.7%. It is important to note that the composition of this formulation was substantially different from ours and the type of container is not specified, so these results cannot be directly compared. Another study about the effect of freezing on cysteamine stability was performed by Pescina et al., in which the effect of pH and the use of penetration enhancers on cysteamine stability were analyzed [42]. They confirmed that the preparations with acid pH, disodium edetate (EDTA) and sodium phosphate were more stable, being those preserved at −20 °C the ones that preserved a higher percentage of cysteamine achieving a stability of up to 24 weeks. Moreover, Bozdağ et al. achieved higher expiration periods (up to 52 weeks), using disodium EDTA and benzalkonium chloride in their composition, with a pH close to 4 [41]. These low pH values are not particularly recommended for chronic use, as it is recommended that these values be as close as possible to the tear pH [43].

Our formulation has a pH of around 6.8, closer to the physiological and, therefore, better tolerated, avoiding irritation and excessive tearing that compromises the permanence of the formulation. These constant values are related to the presence of the sodium citrate and citric acid monohydrate buffer present in the commercial vehicle, AQ [44]. Stability of up to 12 weeks has been achieved by McKenzie et al. using sodium hyaluronate (SH) as a vehicle for cysteamine eye drops when packaged in closed ampoules protected from light at 4 °C. In addition to the differences related to the composition and the method of determination used, the type of packaging proposed would not be the most suitable for the administration of eye drops and their handling. Another alternative proposed to reduce the oxidation of cysteamine was the saturation with nitrogen, although this method has given rise to contradictory results and is not easy to use in the elaboration process [15,26,40].

The COL Eye Drops System used in this work is made of polyvinyl chloride (PVC) and di-n-octyl phthalate/di(2-ethylhexyl) phthalate (DOP/DEHP) free [27], being one of the most widely used materials in the medical field due to its high stability. The effects that freeze-thawing processes may have on the material from which these vials are made are unknown. Therefore, it was previously demonstrated that their elastic properties were not affected, keeping Young’s modulus values constant at different times after freezing [26]. The determination of the oxygen permeability through the walls of the single-dose vials is of relevance given the high oxidizability of cysteamine. Transmission of small molecules of gas, like dry oxygen, through a polymeric material can take place through two processes. First of all, due to the pore effect, in which the gas molecules flow through microscopic pores, pinholes and cracks in the materials. In this case, as demonstrated/mentioned before, the mechanical properties of the material remain stable during the freezing process avoiding the formation of cracks, holes, or tortuous pathways through which the oxygen gas can pass through the polymeric material. Secondly, due to a solubility-diffusion effect, in which the gas molecules dissolved at one of the polymer surfaces diffuse through the polymer to the opposite surface driven by a concentration gradient between the two polymer surfaces. In this case, the concentration difference between the two sides of the polymeric material and the solubility of gases in the corresponding material determines the level of transmission. Both factors, the freezing of the package and the exposure of the polymer to the cysteamine solution over time, prevent the changing the gas solubility in the polymer, consequently, the oxygen transmission values not only did not increase the permeability of the single-use vial to oxygen, but slightly decreased it. The exposure of the polymer to the cysteamine solution also has no effect on the structure of the material under test conditions. It is also important to bear in mind that oxygen diffuses worse in the frozen product than in a liquid solution, so that, in addition to the reduction in permeability of the container itself, the amount of oxygen diffusing into the sample can be expected to be lower.

Numerous studies have described how an increase in residence time decreases the rate of drug elimination favoring the bioavailability and efficacy of the formulation [45,46]. Residence time on the ocular surface is related to viscosity [47]. The association between efficacy and the percentage of HA present in artificial tear has been studied in preclinical and clinical studies, concluding that formulations with higher percentages of HA and higher viscosities improved the signs and symptoms of the dry eye disease because the ocular residence time was increased [45,48,49]. The optimum viscosity of an eye drop was established between 15 to 50 mPa·s [50]. Even though there is such a wide range of viscosities allowed, the optimum value must be the one that does not cause discomfort or blurred vision. In this sense, Ntonti et al. concluded that 24.2 mPa·s could be the most optimal value when the vehicle is HA [51]. Biswas et al. described how more aqueous formulations of cysteamine must have more continuous dosages because of their low bioavailability due to the physiological processes of drainage and eye wash [52]. Liang et al. described that the use of a formulation with a vehicle with better rheological properties improves the bioavailability of the drug, decreasing the dosage [53]. In our formulation, viscosity suffered a significant drop in those vials kept in the freezer, results in line with the previous work [26]. The relevance of this behavior was contrasted with preclinical PET studies in which the residence time on the ocular surface was measured [26], leading to the conclusion that the reduction in viscosity did not translate into a shorter ocular biopermanence time.

In order to confirm these data in humans, an indirect measurement of residence on the ocular surface was performed by measuring TMH and NIKBUT after the instillation of the unfrozen eye drops and after 30 and 120 days of freezing. These techniques provide indirect insight into how viscosity affects the residence time of the vehicle on the ocular surface from tear stabilization data [47,54,55]. In our study, it has been shown that even with differences in viscosity, no changes were found in the THM and NIKBUT values in none of the study days compared to the unfrozen formulation. Therefore, we can conclude that freezing for 120 days does not modify the ocular residence time and the bioavailability of the formulation studied. On the other hand, these techniques do not allow a direct comparison of the data with those previously obtained in rats by PET studies, since it is not possible to fit the data to a mono-exponential equation and obtain a half-life on the ocular surface. Regarding the limitations of the study, it is important to note that the TMH and NIKBUT data are related to tear stability and volume, having been used indirectly in the present study as a way of characterizing the permanence of the vehicle on the ocular surface and, therefore, of the formulation. In addition, although environmental conditions were controlled and maintained, it would be ideal to carry out the study in a chamber that maintains constant temperature and humidity conditions for all individuals and days of the study. On the other hand, the effect of diurnal tear variations has been reduced as much as possible by conducting the bioavailability study between 9 a.m. and 12 a.m., which is one of the greatest strengths of this work based on previous publications [32,33,34]. Another limitation of the present study is that the clinical study was carried out in healthy volunteers, although it would be ideal to perform it in patients diagnosed with cystinosis. Longer retention times would favor the pharmacological action of cysteamine in patients who, if left untreated, are expected to suffer disease progression with crystal formation, leading to photophobia, visual impairment and eventually blindness.

To the authors’ knowledge, our study is the first to utilize the Keratograph 5M to assess the ocular residence time of cysteamine eye drops. Even so, these techniques have been used previously with the aim of characterizing the tear of patients with cystinosis treated with the commercial presentation. Tear film osmolarity and tear breakup time have been evaluated to understand the pathophysiology of adverse reactions reported by cystinosis patients treated with topical viscous cysteamine hydrochloride (Cystadrops^®^). In this respect, Sergio et al. analyzed the tear film breakup time (TBUT) using a slit lamp with a cobalt blue filter and a fluorescein strip. The mean TBUT value was 10.2 ± 0.83 s in the treatment group versus 10 ± 0.7 s in the control group (*p* = 0.62) [56].

Finally, it is important to mention that other techniques as optical coherence tomography (OCT), have been used to evaluate the ocular residence of SH. The dynamics of the tear meniscus after the instillation of 0.1, 0.2 and 0.3% SH showed that all formulations provided higher TMH, being the time with the highest TMH values at 10 min for 0.3% SH, coinciding with the highest viscosity value. They concluded that TMH increased directly with the viscosity and SH concentration, resulting in longer residence times and higher bioavailability [57]. Akiyama-Fukuda et al. evaluated with OCT how the instillation of different HA concentrations affected tear meniscus dynamics by measuring volume, area and height at different times after administration [58]. Tear meniscus measurements were found to be higher with the highest HA concentration, having a bioavailability of up to 15 min. Another study in an animal model of dry eye disease found that animals treated with HA eye drops had higher TBUT values than those treated with BSS^®^. They related the rheological properties of the eye drops with an improvement in tear stability and longer residence times [48].

## 5. Conclusions

Cysteamine hydrochloride eye drops 0.55% in HA packaged in single-dose containers can be stored for 4 months at −20 °C. To ensure its concentration during use, it is recommended to use it at room temperature for the first 16 h after opening. Ocular surface permanence studies in healthy volunteers allow us to conclude storage in freezing for long periods of time does not affect the bioavailability of the formulation or the physical characteristics of the container. These results will reduce the number of visits to the hospital by patients, improving patients’ quality of life, and lightening the workload of the HPD.

## Figures and Tables

**Table 1:** Experimental design of the studies performed every 30 days within the 120 days of the assay.

	DAY 0	DAY 30	DAY 60	DAY 90	DAY 120
Cysteamine quantification	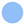	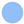	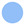	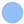	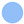
pH and osmolality	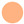	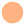	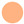	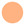	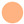
Viscosity	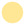	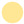			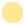
Microbiology	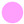		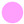		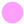
Oxygen permeability	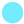	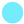			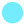
TMH and NIKBUT	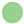	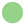			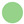

NIKBUT: non-invasive tear keratograph breakup time; TMH: tear meniscus height.

## Data Availability

Not applicable.

## Supplementary Materials

The following supporting information can be downloaded at: https://www.mdpi.com/article/10.3390/pharmaceutics15112589/s1, Table S1. Inclusion and exclusion criteria for participation in the study; Table S2. Descriptive statistics of Tear Meniscus Height (Mean and Sd displayed for parametric variables). Day 0: unfrozen formulation; Day 30: 30-day frozen formulation; Day 120: 120-day frozen formulation; and Table S3. Descriptive statistics of noninvasive keratograph tear breakup time (Mean and SD displayed for parametric variables). Day 0: unfrozen formulation; Day 30: 30-day frozen formulation; Day 120: 120-day frozen formulation.
