# Supplementary material for: Cysteamine Eye Drops in Hyaluronic Acid Packaged in Innovative Single-Dose Systems, Part II: Long-Term Stability and Clinical Ocular Biopermanence
Source: Pharmaceutics. 2023 Nov 5;15(11):2589. doi: 10.3390/pharmaceutics15112589 (PMC10675239; doi:10.3390/pharmaceutics15112589)
Supplement: Supplementary file 1 [file pharmaceutics-15-02589-s001.zip › pharmaceutics-2661454-supplementary.pdf]

Supplementary Material: Cysteamine Eye Drops in Hyaluronic Acid Packaged in Innovative Single-Dose Systems, Part II: Long-Term Stability and Clinical Ocular Biopermanence

Ana Castro-Balado, Andrea Cuartero-Martínez, Hugo Pena-Verdeal, Gonzalo Hermelo-Vidal, Anja Schmidt, Belén Montero, Manuela Hernández-Blanco, Irene Zarra-Ferro, Miguel González-Barcia, Cristina Mondelo-García, María Jesús Giráldez, Eva Yebra-Pimentel, Francisco J. Otero-Espinar and Anxo Fernández-Ferreiro

Table S1. Inclusion and exclusion criteria for participation in the study.

**Inclusion criteria**
Age between 18-40 years
Signed informed consent
DEQ5 <6
Negative staining or 1 with fluorescein or indocyanine green
**Exclusion criteria**
Active ocular pathologies or history of conjunctival, scleral, and corneal disease
Active ocular treatment
Allergies or sensitivities to eye drop components.
Previous ocular surgeries within the last year or general surgeries that may affect the measurements
Use of contact lenses within the last 24 hours
Regular use of medical devices for eye care (artificial tears in the last 24 hours)
Diagnosis of dry eye disease
Symptoms or signs of dry eyes
Thyroid problems
Diabetes mellitus

DEQ5: Five-item Dry Eye Questionnaire

Table S2. Descriptive statistics of Tear Meniscus Height (Mean and Sd displayed for parametric variables). Day 0: unfrozen formulation; Day 30: 30-day frozen formulation; Day 120: 120-day frozen formulation.

**Time (min)**	Mean ± sd (mm) Day 0	Mean ± sd (mm) Day 30	Mean ± sd (mm) Day 120
Baseline	0.293 ± 0.072	0.285 ± 0.055	0.262 ± 0.069
2	0.474 ± 0.090	0.449 ± 0.157	0.420 ± 0.121
5	0.372 ± 0.099	0.396 ± 0.116	0.353 ± 0.098
10	0.324 ± 0.079	0.322 ± 0.091	0.315 ± 0.091
15	0.300 ± 0.064	0.302 ± 0.068	0.284 ± 0.076
30	0.279 ± 0.078	0.284 ± 0.059	0.234 ± 0.043

Table S3. Descriptive statistics of noninvasive keratograph tear breakup time (Mean and SD displayed for parametric variables). Day 0: unfrozen formulation; Day 30: 30-day frozen formulation; Day 120: 120-day frozen formulation.

**Time (min)**	Mean ± sd (mm) Day 0	Mean ± sd (mm) Day 30	Mean ± sd (mm) Day 120
Baseline	10.89 ± 3.498	11.69 ± 6.228	13.14 ± 5.690
2	15.31 ± 6.292	15.65 ± 5.083	14.36 ± 5.001
5	12.92 ± 4.412	14.86 ± 5.686	13.62 ± 5.707
10	12.03 ± 6.076	12.09 ± 4.734	11.88 ± 5.051
15	11.28 ± 4.240	11.20 ± 4.728	11.97 ± 6.200
30	10.67 ± 4.301	11.23 ± 4.526	9.977 ± 5.919
